# Dissociable contributions of frontal and temporal brain regions to basic semantic composition

**DOI:** 10.1093/braincomms/fcab090

**Published:** 2021-04-23

**Authors:** Astrid Graessner, Emiliano Zaccarella, Angela D Friederici, Hellmuth Obrig, Gesa Hartwigsen

**Affiliations:** 1 Lise-Meitner Research Group Cognition and Plasticity, Max Planck Institute for Human Cognitive and Brain Sciences, 04103 Leipzig, Germany; 2 Department of Neuropsychology, Max Planck Institute for Human Cognitive and Brain Sciences, 04103 Leipzig, Germany; 3 Clinic for Cognitive Neurology, University Leipzig, 04103 Leipzig, Germany; 4 Department of Neurology, Max Planck Institute for Human Cognitive and Brain Sciences, 04103 Leipzig, Germany

**Keywords:** meaning composition, lesion-behaviour mapping, SVR-LSM

## Abstract

Semantic composition is the ability to combine single words to form complex meanings and is an essential component for successful communication. Evidence from neuroimaging studies suggests that semantic composition engages a widely distributed left-hemispheric network, including the anterior temporal lobe, the inferior frontal gyrus and the angular gyrus. To date, the functional relevance of these regions remains unclear. Here, we investigate the impact of lesions to key regions in the semantic network on basic semantic composition. We conducted a multivariate lesion-behaviour mapping study in 36 native German speaking participants with chronic lesions to the language network after left-hemispheric stroke. During the experiment, participants performed a plausibility judgement task on auditorily presented adjective-noun phrases that were either meaningful (‘anxious horse’), anomalous (‘anxious salad’) or had the noun replaced by a pseudoword (‘anxious gufel’), as well as a single-word control condition (‘horse’). We observed that reduced accuracy for anomalous phrases is associated with lesions in left anterior inferior frontal gyrus, whereas increased reaction times for anomalous phrases correlates with lesions in anterior-to-mid temporal lobe. These results indicate that anterior inferior frontal gyrus is relevant for accurate semantic decisions, while anterior-to-mid temporal lobe lesions lead to slowing of the decision for anomalous two-word phrases. These differential effects of lesion location support the notion that anterior inferior frontal gyrus affords executive control for decisions on semantic composition while anterior-to-mid temporal lobe lesions slow the semantic processing of the individual constituents of the phrase.

## Teaser

In a lesion-behaviour mapping study, Graessner et al. found dissociable contributions of left-hemispheric brain regions to basic semantic composition: While the anterior inferior frontal gyrus is relevant for task accuracy, the anterior temporal lobe is crucial for efficient semantic processing.

## Graphical Abstract

**Graphical Abstract:**
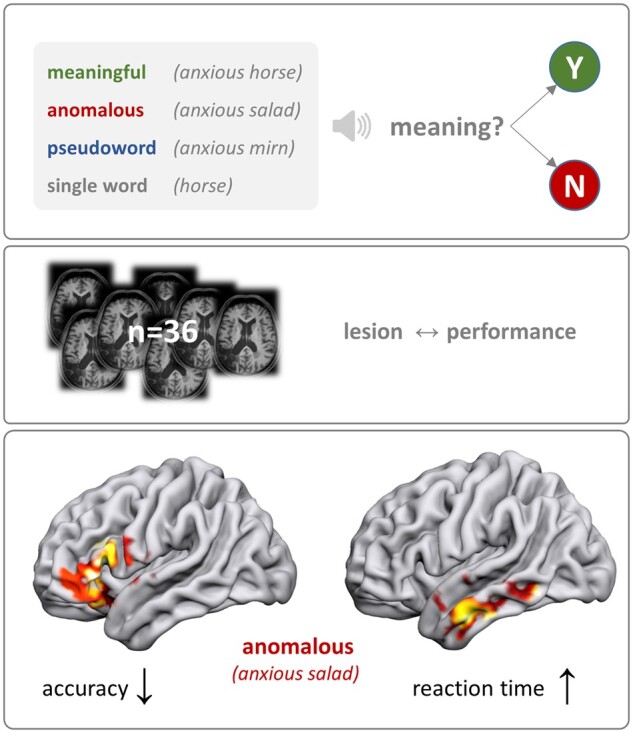

## Introduction

Language comprehension requires rapid mapping of arbitrary word-forms to meaning. For speech comprehension, listeners match the auditory input to stored representations of words and recover the communicative intent in a framework of pre-established world knowledge. Semantic composition is crucial to this process as it allows for the combination of single word meanings to form complex representations.

At the neural level, a widely distributed left-lateralized fronto-temporo-parietal network has been identified for semantic composition. It comprises the anterior temporal lobe (ATL), posterior middle temporal gyrus (pMTG), anterior inferior frontal gyrus (aIFG) and the angular gyrus (AG).^1–7^ To isolate the core combinatorial semantic processes from other cognitive processes during sentence and auditory word comprehension, recent studies used paradigms with very basic two- or three-word phrases. Results converge on a strong contribution of left ATL, aIFG and AG to basic semantic composition.^8–11^ However, the respective functional relevance of these regions for semantic composition remains unclear.

Complementary to functional imaging studies, a powerful approach to investigate the functional anatomical organization of linguistic competence rests on the behavioural assessment in participants with an acquired brain lesion. Such lesion-behaviour mapping in people with post-stroke aphasia has identified crucial regions for language comprehension.^12–17^ In line with the above-described neuroimaging studies, lesion studies confirm the role of a widespread left-hemispheric network including the aIFG, ATL, pMTG and AG to accomplish successful language comprehension.^12^^,^^18–22^ Neurolinguistically, this clearly contradicts early proposals of a singular key role of the posterior superior temporal gyrus (pSTG or ‘Wernicke’s area’) in language comprehension.^14^^,^^23^ Moreover, patholinguistically, the assumption of a widespread network may provide a better framework to explain the recovery from language deficits after damage of a single ‘hub’ in the network, since recovery could rely on preserved neuronal resources.

Previous lesion studies mainly examined deficits of comprehension at the single word or sentence level. This obscures the differentiation between the basic ability of semantic *composition* from lexico-semantic competence and the overall extraction of meaning. On the contrary, simple two-word paradigms should allow us to disentangle the core combinatorial process and aspects of lexical access and lexico-semantic mapping of single entries. Our study is experimental in nature, however, people with no or residual aphasia (as assessed by standard clinical tools) may well struggle with fast semantic composition. Since even slight impairments may interfere with efficient language comprehension, the material used here may serve as a starting point to develop tools to better grasp such aspects of communication in people with an acquired brain lesion.

With respect to the neural correlates of the combinatorial process, we investigate the functional relevance of key semantic regions in the left hemisphere for successful meaning composition by means of multivariate lesion-symptom mapping (LSM) in a cohort of post-stroke language-impaired patients. We probe the ability to judge two-word phrases with regard to the plausibility to form a semantic entity presenting meaningful phrases (‘anxious horse’), anomalous phrases (‘anxious salad’), and pseudoword phrases containing pseudonouns (‘anxious gufel’; see Methods section for details). The design targets the contribution of different brain regions to successful meaning composition by comparing meaningful phrases to phrases in which meaning composition is attempted but should fail (anomalous phrases). To control for overall lexical and executive processes, phrases containing a pseudoword (pseudoword phrases) and a single word condition are included. All two-word phrases are matched on the syntactic and acoustic dimension. The single word comprehension condition serves as low-level baseline for the processing of auditory verbal input and is included to balance forced choice probabilities. We apply multivariate LSM because recent advances in the methodology of LSM have shown that multivariate methods (e.g. support-vector-regression) provide more rigorous and sensitive approaches to associate brain lesions with specific behavioural deficits over classical univariate analyses.^24^^,^^25^

Regarding candidate hubs within the network for semantic processing and meaning composition, neuroimaging and lesion studies speak for a critical role of *ATL*,^8^^,^^26–28^ although the relevance of intact left ATL function has selectively been demonstrated for single words so far.^20^^,^^21^ Beyond ATL, a neuroimaging study in healthy volunteers using the exact same material we apply here, disclosed the *left AG* as another central region for basic semantic composition.^29^ If lesions in this area hamper meaning evaluation of the two-word phrases but not single word retrieval, this would corroborate the central role of left AG proposed by functional neuroimaging. Our hypothesis was that lesions in left AG correlate with impaired judgement of meaningful phrases. Less consistently, the *left aIFG* has also been implicated in semantic processing. The few lesion studies that reported an association of aIFG lesions and language comprehension investigated single word understanding.^19^^,^^22^^,^^30^ Neuroimaging studies, however, suggest that this region is recruited not only during single word comprehension, but contributes to the retrieval of words to be integrated into context.^10^^,^^29^ Consistent with its assumed role in executive semantic processing,^31^^,^^32^ we expected aIFG lesions to hamper judgement of anomalous phrases, which are challenging since the attempt to integrate two meaningful words must be deemed to fail. In summary, our study should provide a comprehensive characterization of the functional relevance of left-hemispheric language areas for basic semantic composition, based on the comparison between meaningful and non-meaningful two-word phrases. Two additional conditions controlled for general lexical access (pseudoword-phrases and single words). Note that the single word condition represents a low-level baseline since judging single existing words as meaningful bears minimal demands with respect to the other conditions included in our design.

## Materials and methods

### Participants

Forty-two native German speaking participants with an acquired chronic left-hemispheric lesion were recruited from the database of the Max Planck Institute for Human Cognitive and Brain Sciences and the Clinic for Cognitive Neurology, University Hospital Leipzig. Exclusion criteria were severe overall cognitive impairment and pre-morbid left-handedness. Six participants had to be excluded (*n* = 1 due to no diagnosed aphasia, *n* = 1 due to additional large right-hemispheric lesion, *n* = 4 due to behavioural results, see Supplementary Figure 1). The group that entered the final analyses consisted of 36 participants [12 females, mean age ± SD (range) = 57 years ± 8 (32–72), mean months since onset = 75 ± 62 (6–291)]. All lesions were of vascular origin including ischaemic and haemorrhagic infarction. It should be noted that the selective inclusion of ischaemic stroke patients is often considered the best choice. However, since ischaemic strokes result from a heterogeneous underlying pathology (e.g. cardiogenic versus generalized angiopathy) and show preferential affection of specific vascular territories, a broader spectrum of lesion sites and aetiologies should attenuate this problem. A general caveat of all lesion-behaviour approaches based on vascular pathologies results from the fact that a large proportion of such patients show ‘unspecific’ white matter lesions, whose potential functional significance reduces the straightforward lesion-behaviour assignment. The mild to moderate aphasia profiles included a large range of aphasia types and severities as classified by Aachener Aphasia Test^33^: Broca’s (*N* = 7), Amnestic (*N* = 4), non-classifiable (*N* = 4), Global (*N* = 3), Wernicke’s (*N* = 1) and Residual (*N* = 17). Participants with residual aphasia at the time of testing had presented with overt aphasia in the acute phase of the disease. Detailed demographic information of the final cohort can be found in Table 1. All participants gave their written informed consent and were financially compensated for their effort. The study protocol conformed to the principles of the Declaration of Helsinki and was approved by the local ethics committee at the University of Leipzig (reference 251/18-ek).

**Table 1:** Patient demographics

Sex	Age	Aetiol	hem	MSO	Aphasia	TT (errors)	Lesvol (cm³)	LeMo LexDec	LeMo Syn	NVST (%)
M	55	Isch	L	20	Non-fl./AoS	6	130.062	66/80	36/40	
M	72	Isch	L	44	Non-fl./AoS		42.050	76/80	38/40	97
F	56	SAH/Isch	L	85	fluent		33.318	78/80	38/40	97
M	52	Isch	L	83	Residual	0	84.355	71/80	22/40	100
M	65	Isch	L	157	Broca	5	164.920	69/80	37/40	100
M	60	Isch	L	107	Residual	0	37.280	77/80	36/40	97
M	58	Isch	L	291	Broca		162.203	80/80	31/40	97
M	51	SAH/Isch	L	42	Residual	2	29.139	79/80	36/40	100
M	56	Isch/TBI	L	57	Residual	0	41.194	74/80	30/40	83
M	48	ICH	L	57	Amn	11	15.893	69/80	33/40	100
F	60	ICH	L	30	Residual	0	10.727	78/80	36/40	83
F	57	Isch	L	73	NCL/AoS/FAS	44	426.250	74/80	35/40	
M	43	Isch	L	59	Residual	1	32.577	79/80	35/40	96
M	60	Isch	L	14	Global		124.042	73/80	38/40	96
M	59	Isch	L	9	Amn	23	83.926	76/80	35/40	96
M	62	Isch	L> R	31	Global		287.825			
M	64	Isch	L	27	Global	29	144.941	71/80	31/40	92
M	56	Isch	L	14	Non-fl./Broca		40.383	74/80	31/40	96
F	67	Isch	L	146	Residual		38.049	77/80	37/40	92
F	53	Isch	L	11	Residual		60.757	75/80	34/40	100
F	66	Isch	L	205	Residual	2	149.943	71/80	35/40	100
F	49	Isch	L	128	Residual		7.724	76/80	37/40	96
M	48	Isch	L	63	NCL	23	46.460	73/80	32/40	97
F	67	ICH	L	54	Residual	0	9.933	79/80	36/40	96
M	55	Isch	L	73	Residual	1	18.529	76/80	40/40	100
M	56	Isch	L	194	Residual	0	81.421	76/80	35/40	100
F	57	Isch	L	75	Broca/Amn	9	53.638	76/80	30/40	92
M	61	Isch	L	6	Amn	10	22.144	73/80	36/40	96
M	59	ICH	L	90	NCL	13	37.855	70/80	34/40	96
M	57	Isch	L	116	Residual	3	38.813	72/80	29/40	100
M	59	Isch	L >R	35	NCL/non-fl.	19	33.649	70/80	34/40	100
F	64	Isch	L	47	Residual	0	24.403	77/80	37/40	96
F	32	SAH	L	50	Non-fl./Broca		90.943	69/80	30/40	100
M	55	Isch	L	12	Residual	0	260.004	73/80	36/40	100
F	62	SAH/Isch	L	128	Amn	21	121.952	76/80	36/40	92
M	48	Isch	L	54	Residual	0	9.391	72/80	37/40	96
12W	Mean **56.9**			**74.6**		**8.54**	**83.2415**	**74.14**	**34.37**	**96.33**
24M	SD **7.5**			**63**		**11.48**	**89.7162**	**3.47**	**3.47**	**4.31**

amn, amnestic; AoS, apraxia of speech; Aetiol, aetiology; hem, lesioned hemisphere; ICH, intracerebral haemorrhage; Isch, ischaemia; LeMo Lex Dec&Syn, absolute scores in the lexical decision and synonym judgement task of the LeMo diagnostics; Lesvol, Lesionvolume in cm^3^; MSO, months since onset; NCL, non-classifiable; non-fl., non-fluent; NVST, percent correct in the nonverbal semantic test; SAH, subarachnoidal haemorrhage; TBI, traumatic brain injury; TT, age-corrected errors in Tokentest > 5 indicates aphasia according to Aachen Aphasia Test metrics.

### Experimental paradigm

**Figure 1:**
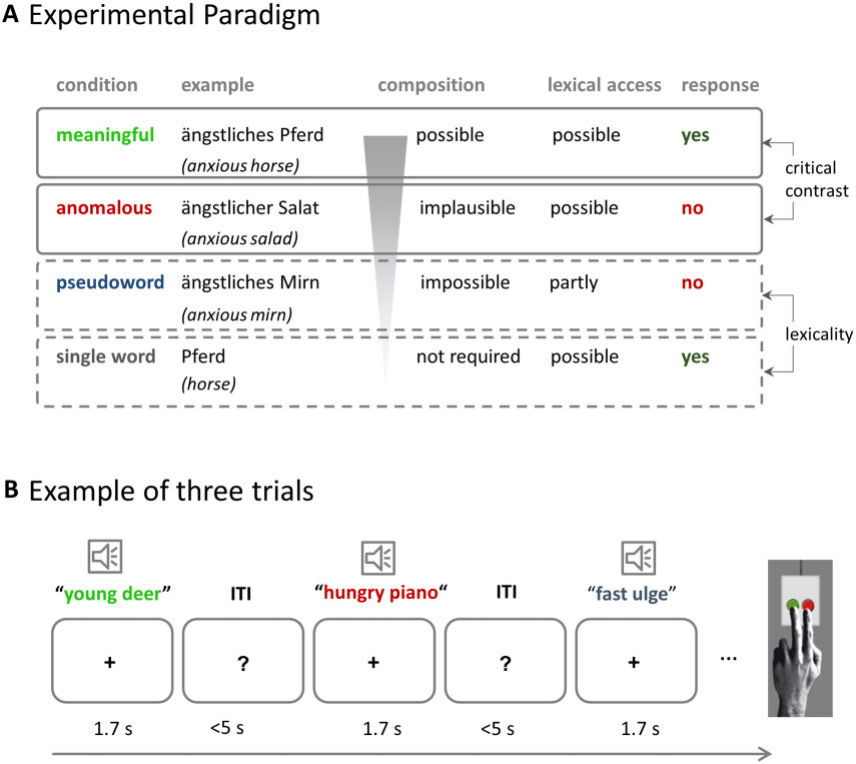
**Experimental design**. (**A**) Experimental conditions with examples, including their function in the experiment and expected responses. (The ‘partly’ for lexical access of pseudoword phrases refers to the adjective). (**B**) Example of three trials.

All participants performed a behavioural experimental paradigm for about 30 min and underwent an additional hour of diagnostic testing and questionnaires (see clinical assessment and Table 1). In the experimental paradigm, auditory stimuli were presented, and participants were asked to judge the meaningfulness of each phrase by forced choice button press (meaningful/not meaningful). Stimuli consisted of spoken word pairs that were either meaningful (‘anxious horse’), anomalous (‘anxious salad’) or had the noun replaced by a pseudoword (‘anxious gufel’). Additional single word stimuli (‘horse’) served as low-level baseline and were included to balance responses, so that 50% of the stimuli required a ‘meaningful’ and 50% a ‘not meaningful’ response. Since we are interested in semantic composition, the critical comparison is between *meaningful* and *anomalous* phrases. The two other conditions control for lexicality judgements (word versus pseudoword) and general executive demands (single word versus two-word phrases. See supplement for more details). Figure 1A illustrates the conditions and their role in the experiment. A practice block checked for comprehension of task requirements. Participants were instructed to respond as quickly and as accurately as possible. Timeout for responses was after 5 s. Subjects gave their response via button press of the left index or middle finger; response button assignment was counterbalanced across participants. The experimental session consisted of eight blocks with all conditions appearing seven times in each block. Stimulus order was pseudo-randomized across participants (56 trials per condition). Blocks were separated by 20-second rest periods. Stimuli were presented using the software Presentation (Neurobehavioral Systems, Inc., Albany, CA, USA).

### Stimuli

The stimuli were identical to those used in a previous fMRI study in neurotypical young volunteers.^29^ The material includes four conditions: (i) The *meaningful* condition (e.g. ‘anxious horse’) allows for successful meaning composition; (ii) The *anomalous* condition comprises two meaningful words which cannot be semantically integrated based on world knowledge (e.g. ‘anxious salad’), since the adjective violates the selectional restriction criteria of the noun (e.g. ‘anxious’ cannot be mapped onto non-living entities). This condition triggers the attempt of meaning composition which should fail in case the lexico-semantic system is intact; (iii) For the *pseudoword* condition, the noun was replaced by a pseudoword (‘anxious gufel’). Note that the syntactic information is kept constant for these three conditions. This avoids confounds by a different number of words or differences in syntactic complexity and allows for largely selective variation of the amount of conceptual/semantic information. (iv) The *single word* condition was included as low-level baseline and to grant equal numbers of ‘meaningful’ and ‘not meaningful’ judgements. The final set of stimuli consisted of 56 phrases per condition, matched for word frequency, orthographic neighbourhood, length, gender and concreteness (for more details see Supplementary Table 1 and Graessner et al.^29^).

### Clinical assessment

To exclude severe deficits in lexical and semantic competence, clinical tests were performed in most participants. This included lexical decision and synonym judgement tasks (*n* = 35, Lexikon Modellorientiert battery^34^), the token test of the Aachen Aphasia Test (*n* = 26) and the standard German non-verbal semantic test (*n* = 33).^35^ The lexical decision task aimed to control for severe comprehension deficits already at the single word level. The synonym judgement task was supposed to test the verbal semantic system at the single word level. Finally, the non-verbal semantic task aimed to guarantee that participants had no general semantic deficit that extended to the non-verbal domain. The token test is a measure of overall aphasia severity. In all other participants, previous clinical testing indicated neither severe lexico-semantic nor non-verbal semantic deficits.

### Structural imaging and lesion delineation

For all participants (*n* = 36), structural imaging was available. These consisted of 30 scans obtained in the in-house MRI scanners (3 T Siemens MRI system Trio^®^ or Verio^®^ system, Siemens Medical Systems, Erlangen, Germany) and included 3D T_1_-weighted- (1 mm isovoxel), and FLAIR-images. In three patients, clinical MRI-imaging at a lower resolution (3–5 mm slice thickness, including FLAIR or TIRM and T_1_ images) was available; in three patients, a cerebral Computed Tomography was used for lesion delineation. Lesions were manually delineated by an experienced neurologist (H.O.) in all three planes on each slice of the T1 or cerebral Computed Tomography-images using MRIcron,^36^ for MRI FLAIR/TIRM-images served as a reference. Images were then transformed into standard stereotactic space (MNI) @1 mm^3^ using SPM12 (www.fil.ion.ucl.ac.uk/spm) and the ‘clinical toolbox’ (nitrc.org/projects/clinicaltbx/), which allows for normalizing images from different modalities into the same space. The unified segmentation approach^37^ was applied and estimation of normalization parameters was restricted to healthy tissue using predefined lesion masks.^38^

### Behavioural analysis

We calculated the mean percentage of correctly answered trials per participant. All reaction times that deviated more than 3 SD from the mean per participant and condition were excluded (1.25% of all trials).

Statistical analyses were performed in R (version 3.6.1) with the generalized linear mixed-effects model using the lme4 package,^39^ assuming a Gamma distribution of our reaction time data. Although our reaction time data were not normally distributed, we followed the advice by Lo & Andrews,^40^ to avoid data transformation for the behavioural analyses, as the generalized linear mixed model accounts for the specific distribution instead of assuming normality. For the analysis of accuracy, we computed a mixed logit regression. We included by-participant intercepts to account for overall inter-individual differences and by-participant slopes. Additionally, we modelled by-item intercepts. To determine statistical significance between each pair of conditions, we used the ‘multcomp’ package.^41^

### Multivariate voxel-based LSM

To investigate lesion-behaviour relationships, we performed multivariate LSM using support-vector-regression (SVR)^25^ as implemented in the SVR-LSM toolbox by DeMarco & Turkeltaub^24^ running under MATLAB R2017b. The advantage of SVR-LSM over classical mass univariate approaches is that it takes inter-voxel correlation into account as it estimates the lesion-symptom map at all voxels simultaneously in a single model. It is thereby less vulnerable to lesion mislocalization and more sensitive to nonlinear relationships.^25^ Another advantage of the SVR-LSM toolbox is that it provides several methods for controlling for overall lesion size. In general, larger lesions lead to more severe behavioural impairments regardless of the lesion location. We controlled for lesion volume by regressing the lesion volume out of both the behavioural scores and the lesion maps, as this method has been shown to provide highest sensitivity.^24^ Only voxels lesioned in at least 10% of participants (4 participants) were included in the analyses. As the raw reaction time data were not normally distributed, we log transformed it before running the lesion analyses.

We ran in total six SVR-LSM analyses to identify lesioned regions associated with lower accuracy in meaningful, anomalous and pseudoword phrases and lesions associated with higher log reaction times in the three conditions. To isolate effects of each condition, we covaried all other conditions out of both the behavioural data and the lesion data using a nuisance model.^24^

Voxel-wise statistical significance was determined by permuting the behavioural scores and randomly reassigning them to participants. SVR-β-value maps were generated for 5000 permutations and thresholded at *P* < 0.005. Although SVR-LSM considers all voxels simultaneously in a single model, statistical significance is determined for each voxel separately, eliciting the well-known problem of multiple comparisons. We thus corrected for multiple comparisons by applying a familywise error rate at a threshold of *P* < 0.05 using a cluster-extent threshold determined from the same 5000 permutations. This method is currently considered the gold standard for lesion symptom mapping.^24^

### Data availability

The data that support the findings of this study are available from the corresponding author, upon reasonable request.

## Results

### Behavioural results

We conducted (generalized) linear mixed-effects models to evaluate behavioural differences between conditions for both reaction times and accuracy. Overall, mean accuracy was high (95.33%), showing that participants were able to perform the task. Large inter-individual differences, however, show that our participants exhibit a wide variety of deficit severity, which is an important factor in identifying lesion-behaviour relationships.^42^

**Figure 2:**
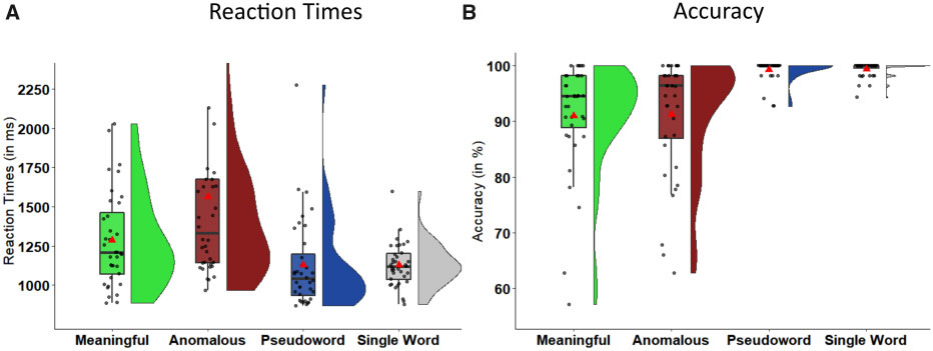
**Behavioural results**. Raincloud plots illustrating the data distribution of individuals’ mean reaction times (left) and accuracy (right) scores and boxplots (including the median as a horizontal line within the box and the first and third quartile as the box’s boundaries) overlaid with individual mean data points for each condition. The red triangle depicts grand mean across participants.

Pairwise post-hoc comparisons between all conditions revealed that reaction times differed significantly between all conditions, with anomalous phrases being processed slowest and single words fastest (Fig. 2A; Table 2). Accuracy was significantly higher for both pseudowords and single words when compared to the two real-word phrasal conditions (Fig. 2B), suggesting that participants were not strongly impaired regarding lexical decision. A detailed list of mean, SD and range for accuracy and reaction time data can be found in Supplementary Table 4. Individual differences from four outliers who performed far below chance level in the anomalous condition are presented in Supplementary Figure 1.

**Table 2:** Behavioural results

Predictor	Reaction times				Accuracy			
	*Coef. ß*	*SE(ß)*	*z*	*P*	*Coef. ß*	*SE(ß)*	*z*	*P*
Accuracy								
ANOM—MEAN	404.392	7.131	56.706	**<0.0001**	0.227	0.399	0.568	1
PSEUD—MEAN	−196.256	10.424	−18.827	**<0.0001**	4.083	0.945	4.322	**<0.0001**
SING—MEAN	−253.312	5.068	−49.982	**<0.0001**	3.009	0.609	4.944	**<0.0001**
PSEUD—ANOM	−600.648	15.039	−39.939	**<0.0001**	3.857	0.912	4.230	**<0.0005**
SING—ANOM	−657.704	9.791	−67.172	**<0.0001**	2.82	0.681	4.083	**<0.0005**
SING—PSEUD	−57.056	9.148	−6.237	**<0.0001**	−1.075	1.099	−0.978	1

Coefficients and *P*-values for post-hoc pairwise comparisons of the linear mixed effects model for reaction time and accuracy. ANOM, anomalous; MEAN, meaningful; PSEUD, pseudowords; SING, single words.

### Lesion-behaviour relationships (SVR-LSM results)

**Figure 3:**
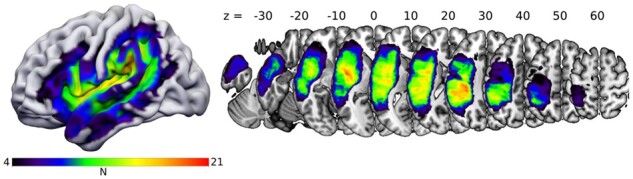
**Lesion overlap map**. The colour scale ranges from 4 lesions (minimum for inclusion in SVR-LSM analyses) to 21 (maximum overlap). Coordinates refer to z-values in MNI-space.

Lesion overlap of the 36 participants is shown in Figure 3. This map shows a broad coverage of language-related regions in the left frontal, temporal and parietal lobe.

**Figure 4:**
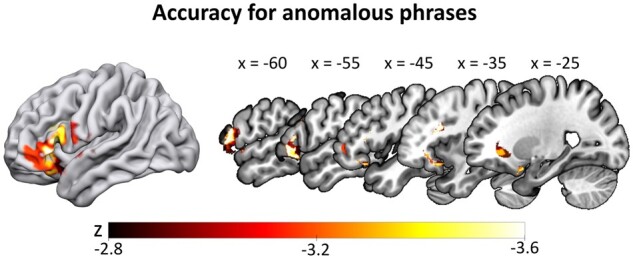
**SVR-LSM results for the accuracy data in the anomalous condition**. Lower accuracy for anomalous phrases (controlled for all other conditions) correlated with a lesion cluster spanning the left inferior frontal gyrus and temporal pole. Thresholded at voxelwise *P* < 0.005 and clusterwise FWE *P* < 0.05 (ranging from *z* = −2.82 to −3.54), with lesion size and the two other conditions regressed out of both behavioural and lesion data.

Our first set of SVR-LSM analyses focussed on lesion correlates of accuracy across the three main conditions, factoring out the impact of the respective other conditions. For anomalous phrases, this revealed a significant cluster in the left IFG (with a peak in pars orbitalis), extending into the temporal pole (clustersize = 12 437 mm³, see Figure 4 and Table 3). It indicates that lesions in this cluster significantly decrease accuracy. All other conditions did not survive multiple comparisons correction.

**Table 3:** Size and location of cluster peaks of the SVR-LSM analyses

Region	Cluster size (mm³)	*x*	*y*	*z*	Peak value (*z*)
Cluster peaks for SVR-LSM of accuracy for anomalous phrases					
IFG/insula/temporal pole	12 437				
IFG (pars orbitalis)		−38	21	−18	−3.54
IFG (pars orbitalis)		−26	12	−23	−3.35
IFG (pars orbitalis)		−29	20	−22	−3.24
IFG (pars orbitalis)		−36	30	−16	−3.16
Temporal pole		−22	8	−27	−3.09
Insula		−31	18	−16	−3.04
Cluster peaks for SVR-LSM of Log RT for anomalous phrases					
ATL/MTG	7367				
ATL (inferior)		−53	−8	−32	−3.54
ATL (middle)		−59	−2	−29	−3.35
ATL (inferior)		−51	−3	−39	−3.16
ATL (inferior)		−56	−9	−36	−3.09
pMTG		−60	−14	−32	−3.04
ATL (middle)		−52	−12	−25	−2.99

All cluster peaks were corrected for the lesion volume.

**Figure 5:**
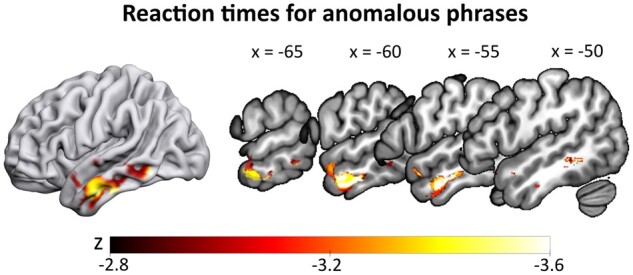
**SVR-LSM results for reaction times in the anomalous condition**. Slower reaction times for anomalous phrases (controlling for all other conditions) correlated with lesions in the left anterior temporal lobe and middle temporal gyrus. Thresholded at voxelwise *P* < 0.005 and clusterwise FWE *P* < 0.05 (ranging from *z* = −2.77 to −3.54), with lesion size regressed out of both behavioural and lesion data.

Reaction times also yielded significant results for anomalous phrases only. Here, lesions in the anterior to posterior temporal lobe, spanning inferior and middle parts (clustersize = 7367 mm³, see Figure 5 and Table 3), were correlated with slower reaction times for anomalous phrases, when factoring out all other conditions.

For validation and comparison of the multivariate results to traditional univariate approaches, we repeated the analyses using the *NiiStat* toolbox for Matlab (https://github.com/neurolabusc/NiiStat). Besides being univariate, a major difference is the method to correct for lesion volume. Regressing lesion volume on the behavioural predictor only has been shown to be overly conservative.^24^ We thus conducted comparable analyses in NiiStat without correcting for lesion size. Overall, these analyses showed the same results as the SVR-LSM analyses and results can be found in the supplementary materials. Additionally, lesion correlates for reaction times showed a small cluster in the left ATL for meaningful phrases.

## Discussion

Incremental integration of semantic units is a prerequisite for comprehension of language and thereby essential for successful everyday communication. Here, we investigated the functional relevance of left hemispheric language areas for efficient and accurate basic semantic composition in a cohort of chronic post-stroke language-impaired participants. Participants performed a meaningfulness judgement task on three types of auditorily presented two-word phrases: meaningful (*anxious horse*), anomalous (*anxious salad*) and pseudoword phrases (*anxious gufel*), as well as on single words (*horse*). Despite their language impairment, participants performed rather accurately on the meaningfulness judgement task, in line with the relatively mild impairment of the tested sample. This may motivate the application of similar material in clinical settings to detail the precise locus of impairment in people with mild or residual aphasia. Judgement on meaningful and anomalous phrases showed large interindividual differences while for pseudoword phrases and single words participants approached ceiling performance. This confirms that the challenge of our material regards the success of meaning composition since these two conditions require a meaningfulness judgement beyond basic lexical abilities. Correlating individual behaviour with lesion site revealed differential effects of lesion site on different aspects of semantic composition. Lesions to left aIFG selectively impaired task accuracy for anomalous phrases. In other words, participants with lesions in this region showed a lower threshold for judging phrases as meaningful, increasing the erroneous acceptance of phrases such as ‘anxious salad’ as meaningful. Conversely, lesions to left ATL/MTG were associated with prolonged reaction times in anomalous phrases. This means that participants with ATL/MTG-lesions needed more time to correctly discard anomalous phrases as meaningless, putatively indicating increased effort in combining separate concepts. Hence our paradigm is sensitive to detect differences in accurate and efficient judgements. Notably, these two aspects of task performance dissociate between two brain regions, which are part of the larger ‘lexico-semantic network’.

Our results show that intact left aIFG function is crucial to accurately set the threshold regarding the overall meaningfulness of a phrase. Note that lesions to this area were associated with increased errors only for the most challenging condition, resulting in misclassifications of anomalous phrases as meaningful. Such a misadaptation of the decision threshold resulting from lesions in the frontal part of the language network has been previously proposed for other aspects of semantic competence.^43^^,^^44^ Our finding provides novel evidence for a crucial role of the left aIFG in executive semantic processing during minimal word combinations. In contrast, lesions to left ATL/MTG were associated with increased response latencies for anomalous phrases, supporting the assigned key role of these areas in conceptual-semantic integration. Based on the observed neuroanatomical dissociation in our data, we propose that accuracy and efficiency of the plausibility judgement might rely on different mechanisms housed in different hubs of the language network.

### The left anterior IFG is crucial for accurate semantic composition

Our finding of a key contribution of the left anterior IFG to semantic plausibility judgements is consistent with numerous neuroimaging and neurostimulation studies arguing for a crucial role of the aIFG in executive semantic control at the word level.^27^^,^^31^^,^^32^^,^^45^ Additional evidence for the role of left IFG in plausibility judgement of minimal phrases comes from Graves et al.,^46^ reporting increased IFG activation for the very uncommon (meaningless) compared to the common order of two-word phrases (e.g. ‘apple tree’ versus ‘tree apple’). Supporting and extending these findings, our study is the first to show that this region is crucial for plausibility judgements of anomalous phrases. Importantly, lesions to aIFG selectively impaired task accuracy but not response speed and did so selectively for anomalous phrases. Accuracy regarding rejection of pseudoword phrases (‘anxious gufel’) showed no correlation with lesions in this area. Setting an overly liberal threshold of acceptance after lesions to the left aIFG would support its pivotal role in processes at the final stage of response selection in challenging linguistic-semantic tasks.^47^^,^^48^ We do not wish to suggest that the role of the left aIFG in semantic processing is restricted to the allocation of executive control. However, the role of this area in making semantic judgements may be crucial when decisions on conflicting input are required (two meaningful words but meaningless combination). Support for this notion comes from the processing of meaningful phrases. These phrases were processed faster than anomalous phrases and the individual accuracy levels did not correlate with lesions in the left aIFG. In sum, we suggest that lesions to the left aIFG lead to a more liberal threshold for considering phrases meaningful, when this judgement implies resolving conflict between meaningful single constituents and the absence of meaning of the phrase.

### The left anterior and middle temporal cortex is crucial for efficient semantic composition

Lesions to the left ATL/MTG correlated selectively with prolonged reaction times for anomalous phrases but did not account for higher error rates. Converging evidence from neuroimaging and neuropsychological studies have identified the bilateral ATL as a core semantic hub.^27^^,^^49^ Particularly, the left ATL is thought to be crucial for the interface between overall semantics and lexico-semantic processes during word production^50–52^ and comprehension.^20^^,^^21^ Interestingly, we do not find ATL lesions to affect word comprehension, since the performance on pseudoword phrases with a real adjective and a pseudo-noun did not correlate with lesions in this region. Notably, our task did not require object recognition; instead, the meaning of two words had to be mapped onto conceptual representations to then check whether the conceptual representation can be merged in the context of pre-existing world knowledge. While word comprehension errors are associated with lesions to more posterior regions in the temporal lobe,^12^ the ATL is rather involved in conceptual semantic processes. Its role in responding to semantic aspects of local phrase structure building^6^ and attention on semantic-syntactic integration^53^ are in line with this view. The posterior parts of the temporal lobe rather afford the mapping of auditory word forms to concepts.^12^ The fact that our results did not show correlations with this important language area supports our view that plausibility judgement on phrases requires semantic rather than word retrieval processes. Word retrieval was no specific challenge to the participants and was relevant for all conditions. Our results thus support the notion that the ATL belongs to the deeper semantic network, while posterior temporal regions may rather link auditory input to concepts. The fact that ATL lesions selectively delayed meaningfulness judgements for anomalous phrases without increasing error rates is likely explained by the bilateral contribution of the ATL to semantic processing.^54^ The unimpaired right ATL or other intact regions of the semantic network might have compensated for left hemispheric lesions during semantic judgements, causing an increased processing time for a nonetheless correct judgement. Future investigations may specifically address these compensatory mechanisms of right ATL after left hemispheric stroke in semantic composition by applying inhibitory neurostimulation to this area.

Contrary to our hypotheses, we did not find an association between lesions in the AG and performance in any condition. A number of fMRI studies have suggested a contribution of left (and sometimes right) AG to basic semantic composition.^10^^,^^11^^,^^29^^,^^46^ Moreover, lesion evidence has shown that bilateral AG atrophy led to a specific meaning composition deficit, over and above single word comprehension.^11^ In contrast, our study did not reveal a significant relationship between AG lesions and basic compositional processes. While this area was well-covered regarding lesion overlap (see Supplementary Figure 4), null findings are generally more difficult to interpret. However, this finding it is still compatible with current theories on the neural correlates of the semantic network. The widespread network affording semantic analysis including semantic composition may be partially redundant when it comes to semantic tasks which are deliberately designed to be simple and necessarily repetitive in a test-situation. The AG undoubtedly is part of the semantic network, but our study speaks for a less relevant role during basic semantic composition. In line with this notion, a previous neurostimulation study showed that perturbing either AG or aIFG alone did not lead to a disruption in semantic word decisions in healthy volunteers, while combined perturbation delayed semantic judgements.^55^ Aside from recruiting other intact nodes from the left-hemispheric semantic network, patients with left AG lesions may also have relied more on their intact right AG during the task^11^; see also Graves et al.^46^

Regarding the methodological approach used in the present paper, we wish to emphasize that univariate control analyses support our claims. In light of recent debates about the superiority of one method over the other, we are confident that the converging evidence from both analyses in our study follows best practice recommendations to exploit specific advantages of either method.^56^

### Limitation and perspectives

Although lesion-behaviour approaches are powerful, and the approach applied here may be considered state-of-the-art, sample size and the fact that lesion-site bias is inherent to homogeneous aetiology samples are notorious challenges. Moreover, stable and chronic lesions naturally imply that substantial compensatory network-reorganization must be assumed. A promising way to address this issue is the combination with reversible functional impairment by neurostimulation.^48^ Additionally, the role of the right hemisphere in semantic processing is clearly only beginning to be experimentally addressed, an avenue which is worth following in future studies.

## Conclusion

Our results provide novel evidence for the differential roles of inferior frontal and anterior/middle temporal cortex in basic semantic composition. We show a division of labour between these key semantic areas at the most basic level of semantic composition and provide evidence for a neural dissociation in the processing of task efficiency and accuracy. Overall, our results may help to establish a sensitive diagnostic measure for basic semantic composition that allows to distinguish between task accuracy and efficiency. Regarding therapeutic intervention, future studies may specifically target learning aspects of the task, including both semantic analysis and decision processes involved in semantic composition. Undoubtedly, improvement of this very basic process will be central for restoring impaired language comprehension.

## Supplementary material

Supplementary material is available at *Brain Communications* online.

## Supplementary Material

fcab090_Supplementary_Data

## Abbreviations

AG =: angular gyrus
aIFG =: anterior inferior frontal gyrus
ATL =: anterior temporal lobe
LSM =: lesion-symptom mapping
NVST =: Nonverbal Semantics Test
pMTG =: posterior middle temporal gyrus
SD =: standard deviation
SVR-LSM =: support vector regression lesion-symptom mapping
