# Supplementary material for: Dissociable contributions of frontal and temporal brain regions to basic semantic composition
Source: Brain Commun. 2021 Apr 23;3(2):fcab090. doi: 10.1093/braincomms/fcab090 (PMC8212833; doi:10.1093/braincomms/fcab090)
Supplement: fcab090_Supplementary_Data [file fcab090_supplementary_data.docx]

**Supplementary Material**

**Supplementary Materials and Methods**

**Supplementary Table 1.** Psycholinguistic variables for the two real-word conditions.

|  | Anomalous | Meaningful | *Statistics* |
| --- | --- | --- | --- |
| **Adjectives** | | | |
| Frequency | 2.13 (0.96) | 2.11 (0.95) | *t*(109.99) = 0.13, *p* = 0.89 |
| OLD-20 | 1.98 (0.45) | 1.91 (0.43) | *t*(109.86) = 0.84, *p* = 0.39 |
| **Nouns** | | | |
| Frequency | 2.56 (0.59) | 2.57 (0.55) | *t*(109.66) = -0.09, *p* = 0.92 |
| OLD-20 | 1.66 (0.35) | 1.67 (0.31) | *t*(108.57) = -0.21, *p* = 0.83 |
| Concreteness | 4.86 (0.13) | 4.85 (0.15) | *t*(108.18) = 0.35, *p* = 0.72 |
| **Pairs** |  |  |  |
| Meaningfulness rating | 1.47 (0.31) | 5.49 (0.36) | ***t*(107.98) = -63.54, *p* < 0.0001** |
|  | *1.28 (0.32)* | *5.59 (0.41)* | ***t*(58.28) = 46.77, *p* < 0.0001** |

Frequency and OLD-20 (orthographic neighborhood) measures were taken from the SUBTLEX-DE database and frequency is given as log-transformed per 1 million words. Concreteness was determined using concreteness ratings for 40.000 English words. Meaningfulness ratings were obtained from 20 healthy participants who did not take part in the experiment. Ratings in italics are averaged ratings of the VLSM participants in a post-hoc questionnaire. They indicate great overlap with our predefined conditions. Numbers in brackets in the first two columns represent standard deviation. Numbers in the statistics column represent degrees of freedom, t-values and p-values as calculated from the welch’s t-test.

**Supplement to *Experimental Paradigm***

A reviewer pointed out that single words were always meaningful and might thereby trigger specific response strategies. Please note that 9 participants did not perform at ceiling in this condition. More accurate performance on meaningful compared to anomalous phrases was relatively balanced across the sample (meaningful > anomalous: n=15; meaningful < anomalous: n=21). We initially considered to fully balance the design, which would have required single pseudowords and two-pseudoword phrases. However, to optimize time constraints and since our focus is on semantic composition rather than lexical access, the more parsimonious design was chosen.

**Supplementary Table 2.** Stimulus list for anomalous and meaningful phrases, including the plausibility rating from an independent subject group.

| **Condition** | **Stimulus (english translation)** | **Mean Plausibility Rating** |
| --- | --- | --- |
| **A** | nervoeser Salat (anxious salad) | 1.05 |
| **A** | aengstliches Holz (anxious wood) | 1.1 |
| **A** | braver Lappen (obedient cloth) | 1.1 |
| **A** | felsiges Schaf (rocky sheep) | 1.1 |
| **A** | fluessiges Auto (liquid car) | 1.1 |
| **A** | gestimmtes Schwein (tuned pig) | 1.1 |
| **A** | ruhiger Krug (quiet jug) | 1.1 |
| **A** | stumpfer Fisch (blunt fish) | 1.1 |
| **A** | zahmes Brett (tame board) | 1.1 |
| **A** | koestliches Schiff (delicious ship) | 1.2 |
| **A** | morsches Kamel (rotten camel) | 1.2 |
| **A** | toter Korb (dead basket) | 1.2 |
| **A** | blinder Finger (blind finger) | 1.25 |
| **A** | frisches Dreieck (fresh triangle) | 1.25 |
| **A** | hoelzerner Schaum (wooden foam) | 1.25 |
| **A** | lautes Auge (loud eye) | 1.25 |
| **A** | modischer Spinat (fashinable spinach) | 1.25 |
| **A** | mutiger Knochen (brave bone) | 1.25 |
| **A** | poroeser Schwan (porose swan) | 1.25 |
| **A** | sportliches Glas (sporty glass) | 1.25 |
| **A** | stolzes Kabel (proud wire) | 1.25 |
| **A** | pikanter Teppich (spicy carpet) | 1.3 |
| **A** | behaarter Ofen (hairy oven) | 1.35 |
| **A** | defekter Loewe (defective lion) | 1.35 |
| **A** | hungriges Klavier (hungry piano) | 1.35 |
| **A** | luftiger Teller (airy plate) | 1.35 |
| **A** | ovales Pferd (oval horse) | 1.35 |
| **A** | eckiges Pferd (rectangular horse) | 1.4 |
| **A** | lockerer Pfau (loose peacock) | 1.45 |
| **A** | niedriger Pinsel (low brush) | 1.45 |
| **A** | defekter Fuchs (defective fox) | 1.5 |
| **A** | einsamer Motor (lonely engine) | 1.5 |
| **A** | leerer Guertel (empty belt) | 1.5 |
| **A** | senkrechter Helm (vertical helmet) | 1.5 |
| **A** | wilder Koffer (wild suitcase) | 1.5 |
| **A** | zackiger Schlauch (pointed tube) | 1.5 |
| **A** | biegsamer Mond (flexible moon) | 1.55 |
| **A** | faules Kleid (lazy dress) | 1.55 |
| **A** | junger Kittel (young smock) | 1.55 |
| **A** | bequemer Zahn (comfortable tooth) | 1.6 |
| **A** | gesunder Spiegel (healthy mirror) | 1.6 |
| **A** | wilder Stuhl (wild chair) | 1.6 |
| **A** | dummer Schal (stupid scarf) | 1.65 |
| **A** | stabiler Wolf (stable wolf) | 1.65 |
| **A** | weiblicher Tisch (female table) | 1.7 |
| **A** | freches Paket (naughty package) | 1.8 |
| **A** | kaputter Schwan (broken swan) | 1.85 |
| **A** | wachsames Plakat (vigilant poster) | 1.85 |
| **A** | sauberer Mond (clean moon) | 1.9 |
| **A** | unscharfes Hemd (blurry shirt) | 1.9 |
| **A** | offenes Fahrrad (open bicycle) | 1.95 |
| **A** | steinerner Brief (stone letter) | 1.95 |
| **A** | stoerrischer Berg (stubborn mountain) | 2.05 |
| **A** | schmales Herz (narrow heart) | 2.1 |
| **A** | eiserner Pilz (iron mushroom) | 2.2 |
| **A** | rostiger Vogel (rusty bird) | 2.25 |
| **M** | niedriger Spiegel low mirrow) | 4.55 |
| **M** | ruhiger Motor (quiet engine) | 4.65 |
| **M** | einsamer Vogel (lonely bird) | 4.75 |
| **M** | felsiger Berg (rocky mountain) | 4.85 |
| **M** | ovaler Teller (oval plate) | 4.85 |
| **M** | spitzes Dreieck (pointy triangle) | 4.95 |
| **M** | braves Schaf (obedient sheep) | 5.1 |
| **M** | senkrechter Pfosten (vertical pole) | 5.1 |
| **M** | gesundes Holz (healthy wood) | 5.15 |
| **M** | pikanter Fisch (spicy fish) | 5.2 |
| **M** | stolzer Pfau (proud peacock) | 5.2 |
| **M** | zackiger Stern (ponted star) | 5.2 |
| **M** | poroeser Knochen (porose bone) | 5.25 |
| **M** | steinernes Kreuz (stone cross) | 5.25 |
| **M** | toter Wolf (dead wolf) | 5.25 |
| **M** | defekter Schlauch (defective tube) | 5.3 |
| **M** | hohler Zahn (hollow tooth) | 5.3 |
| **M** | hoelzernes Schiff (wooden ship) | 5.35 |
| **M** | zahmer Loewe (tame lion) | 5.35 |
| **M** | aengstliches Pferd (anxious horse) | 5.4 |
| **M** | frischer Lappen (clean cloth) | 5.4 |
| **M** | eiserner Ofen (iron oven) | 5.45 |
| **M** | frecher Affe (naughty monkey) | 5.5 |
| **M** | morscher Baum (rotten tree) | 5.5 |
| **M** | sturer Esel (stubborn donkey) | 5.5 |
| **M** | handliches Paket (compact package) | 5.55 |
| **M** | hungriger Fuchs (hungry fox) | 5.55 |
| **M** | koestlicher Salat (delicious salad) | 5.55 |
| **M** | leeres Plakat (blank poster) | 5.55 |
| **M** | luftiges Kleid (airy dress) | 5.55 |
| **M** | weiblicher Schwan (female swan) | 5.55 |
| **M** | biegsames Kabel (flexible wire) | 5.6 |
| **M** | dummes Schaf (stupid sheep) | 5.6 |
| **M** | lautes Auto (loud car) | 5.6 |
| **M** | offener Beutel (open bag) | 5.6 |
| **M** | duenner Pinsel (thin brush) | 5.65 |
| **M** | krummer Finger (crooked finger) | 5.65 |
| **M** | blindes Auge (blind eye) | 5.7 |
| **M** | giftiger Pilz (poiseneous mushroom) | 5.7 |
| **M** | schmaler Guertel (narrow belt) | 5.7 |
| **M** | rostiges Schwert (rusty sword) | 5.8 |
| **M** | sauberes Glas (clean glass) | 5.8 |
| **M** | bequemes Hemd (comfortable shirt) | 5.85 |
| **M** | junger Hirsch (young deer) | 5.85 |
| **M** | leerer Koffer (empty suitcase) | 5.85 |
| **M** | roher Spinat (raw spinach) | 5.85 |
| **M** | runder Mond (round moon) | 5.85 |
| **M** | stabiles Regal (stable shelf) | 5.85 |
| **M** | blutiges Knie (bloody knee) | 5.9 |
| **M** | eckiger Tisch (rectangular table) | 5.9 |
| **M** | gestimmtes Klavier (tuned piano) | 5.9 |
| **M** | kaputtes Fahrrad (broken bicycle) | 5.9 |
| **M** | reifer Kaese (mature cheese) | 5.9 |
| **M** | modischer Schal (fashionable scarf) | 5.95 |
| **M** | bequemer Stuhl (comfortable chair) | 6 |
| **M** | unscharfes Foto (blurry photo) | 6 |

**Supplementary Table 3.** Post-study rating results for 32 participants.

| Subject | Subject Mean Anomalous | Subject Mean Meaningful |
| --- | --- | --- |
| P_01 | 1.04 | 5.91 |
| P_02 | 1.11 | 5.84 |
| P_03 | 1.98 | 5.86 |
| P_04 | 1.16 | 5.55 |
| P_05 | 1.43 | 5.73 |
| P_06 | 1.09 | 5.64 |
| P_08 | 1.18 | 5.93 |
| P_09 | 1.27 | 5.46 |
| P_10 | 1.45 | 5.43 |
| P_11 | 1.09 | 5.89 |
| P_12 | 2.00 | 5.78 |
| P_13 | 1.00 | 5.55 |
| P_15 | 1.96 | 5.43 |
| P_16 | 1.16 | 5.66 |
| P_18 | 1.36 | 3.98 |
| P_19 | 1.45 | 5.73 |
| P_21 | 1.43 | 5.93 |
| P_22 | 1.14 | 5.91 |
| P_24 | 1.07 | 5.50 |
| P_25 | 1.00 | 5.46 |
| P_26 | 1.00 | 5.10 |
| P_27 | 1.20 | 5.98 |
| P_28 | 1.10 | 5.13 |
| P_29 | 1.16 | 5.71 |
| P_30 | 2.16 | 5.82 |
| P_31 | 1.07 | 5.70 |
| P_33 | 1.00 | 6.00 |
| P_34 | 1.44 | 4.79 |
| P_35 | 1.07 | 5.84 |
| P_36 | 1.13 | 5.89 |
| P_39 | 1.25 | 5.48 |
| P_41 | 1.18 | 5.16 |
| MEAN | **1.28** | **5.59** |
| STD | **0.32** | **0.41** |

**Supplementary Results**

***Behavioral results***

**Supplementary Table 4.** Mean, SD and range for accuracy and reaction time data.

| condition | Mean accuracy (%) | SD accuracy (%) | Range (%) | Mean RT (ms) | SD RT (ms) | Range (ms) |
| --- | --- | --- | --- | --- | --- | --- |
| Meaningful | 91.25 | 9.79 | 57.14-100 | 1283 | 524.5 | 462-4647 |
| Anomalous | 91.41 | 10.51 | 62.86-100 | 1524 | 750.8 | 638-4912 |
| Pseudoword | 99.28 | 1.94 | 92.73-100 | 1123 | 376.8 | 595-3784 |
| Single word | 99.39 | 1.34 | 94.29-100 | 1124 | 235.5 | 679-2557 |

**Outliers**: Four participants performed far below chance in the anomalous condition while performance on the other conditions was comparable to all other participants. Their response pattern indicates that they did not understand the task properly and they were excluded from the analyses.


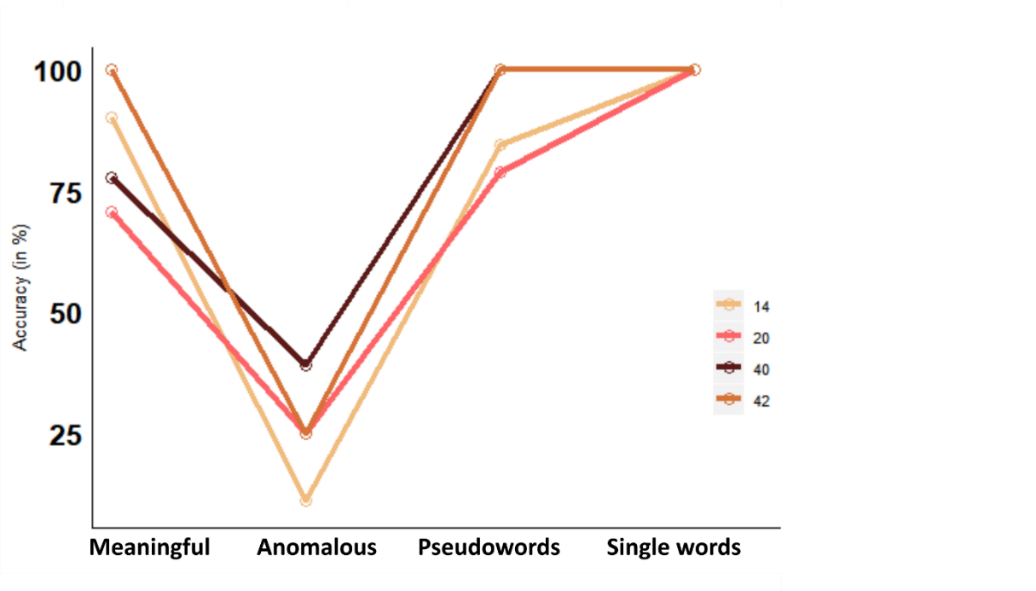


**Supplementary Figure 1**. Accuracy for the 4 excluded participants on all conditions.

***VLSM – Univariate analyses with NiiStat***

To confirm our multivariate analyses, we additionally conducted univariate lesion-behavior mapping analyses using NiiStat (<https://github.com/neurolabusc/NiiStat>). Correspondingly, accuracy and reaction times in the 3 main conditions served as predictors and scores on all other conditions were partialed out as nuisance regressors, using the Freedman-Lane procedure incorporated in NiiStat. The minimum lesion overlap was set to 4 participants, as in the multivariate analyses described in the main text and all analyses were corrected for multiple comparisons via permutation testing (5000 permutations, p < 0.05 FWE).


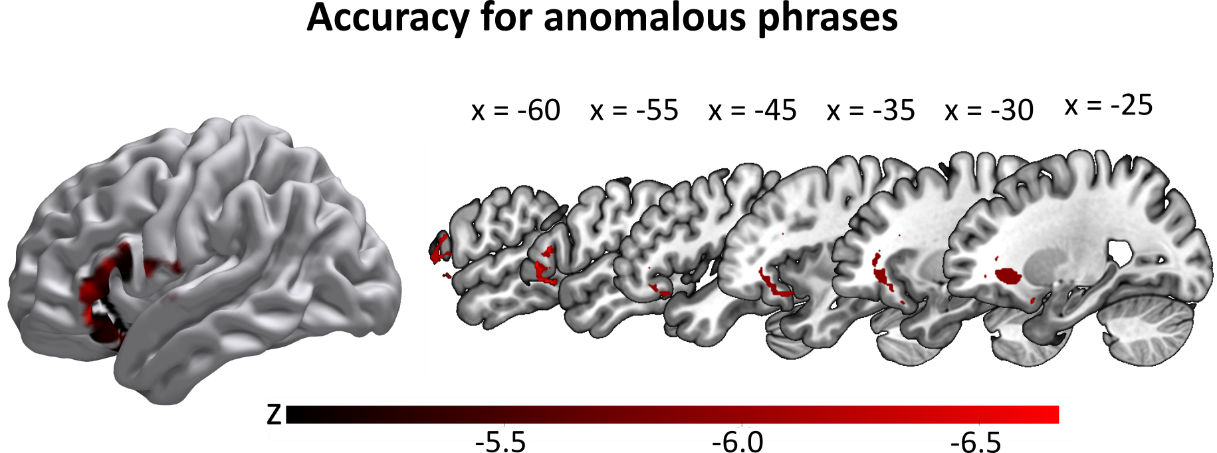


**Supplementary Figure 2**. Univariate results for the accuracy data in the anomalous condition. Lower accuracy for anomalous phrases (controlled for all other conditions) correlated with a lesion cluster spanning the left inferior frontal gyrus. Thresholded at FWE p < 0.05.


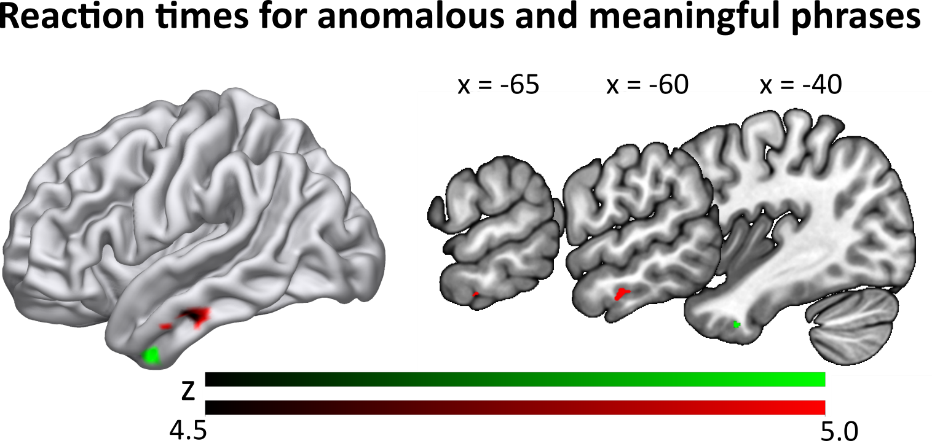


**Supplementary Figure 3.** Univariate results for the reaction time data in the anomalous and meaningful condition. Lower accuracy for anomalous phrases (controlled for all other conditions) correlated with lesions in the left anterior temporal lobe (red cluster) and lower accuracy for meaningful phrases (controlled for all other conditions) correlated with lesions in left anterior temporal pole (green cluster) . Thresholded at FWE p < 0.05.


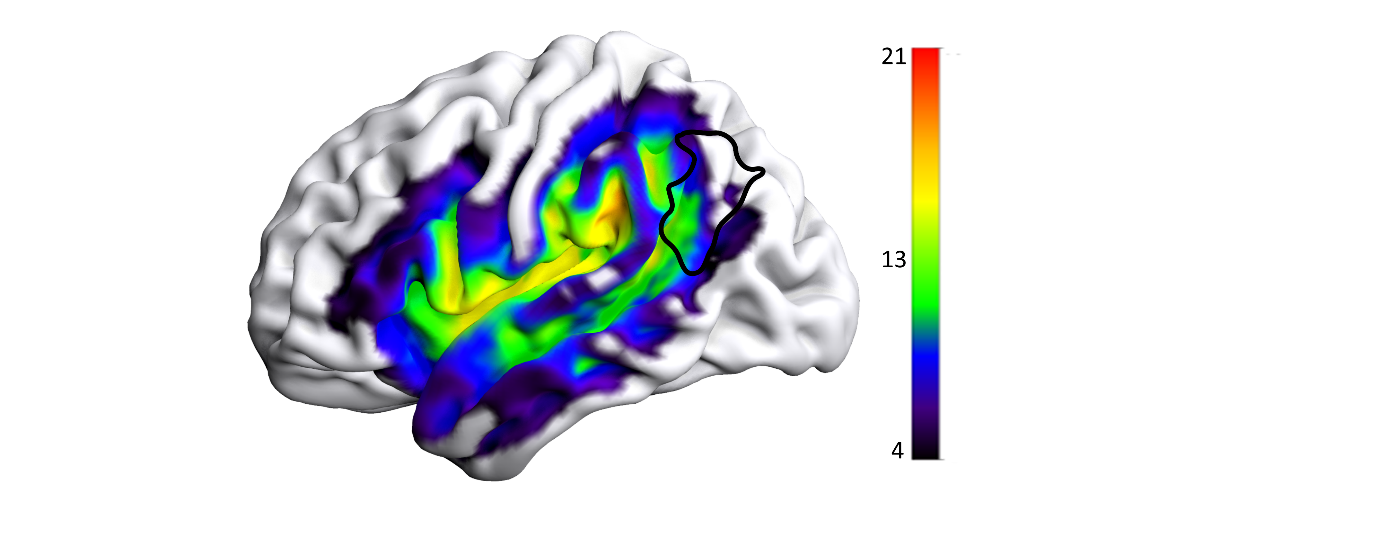


**Supplementary Figure 4**. Lesion overlap map for 36 participants. The color scale ranges from 4 lesions to 21 (maximum overlap). The black outline shows the probability map from the SPM Anatomy toolbox for the cytoarchitectonic region PGa. The maximum overlap in region PGa is 13 participants.
